# Evaluation of practices for the diagnosis and monitoring of cytomegalovirus infection in kidney transplant recipients in Brazil

**DOI:** 10.1590/2175-8239-JBN-2025-0201en

**Published:** 2025-11-28

**Authors:** Nayla Azanki Hatem, Elizete Keitel, Guilherme Santoro-Lopes, Ligia Camera Pierrotti, Alessandro Comarú Pasqualotto

**Affiliations:** 1 Universidade Federal de Ciências da Saúde de Porto Alegre, Programa de Pós-Graduação em Patologia, Porto Alegre, RS, Brazil.; 2 Santa Casa de Misericórdia de Porto Alegre, Porto Alegre, RS, Brazil.; 3 Universidade Federal do Rio de Janeiro, Rio de Janeiro, RJ, Brazil.; 4 Universidade de São Paulo, São Paulo, SP, Brazil.

**Keywords:** Cytomegalovirus, Diagnosis, Therapy, Survey, Kidney transplantation

## Abstract

**Introduction:**

Cytomegalovirus (CMV) remains a significant challenge in kidney transplantation. Despite prophylactic and preemptive antiviral strategies, clinical practices vary widely. This study assessed CMV diagnostic and monitoring practices in Brazilian kidney transplant centers, focusing on access to diagnostic tools, therapeutic thresholds, and logistical barriers.

**Methods:**

A nationwide electronic survey was conducted between August and October 2024, targeting all kidney transplant programs (TP) registered with the Brazilian Society of Organ Transplantation (ABTO).

**Results:**

A total of 35 TP (20.6% response rate) participated, representing 62% of kidney transplants performed in Brazil in 2023. While most centers had CMV management protocols (94.3%), significant variability was observed in the initiation of preemptive therapy (PET). Among high-risk patients (D+/R−), 41.9% followed predefined thresholds. Specific cut-off values were applied in 71.0% of R+ patients and in 45.2% of the low-risk group (D−/R−). Quantitative PCR was the primary diagnostic method (97.1%), with whole blood (60%) and plasma (34.3%) as preferred sample types. A significant proportion of CMV TP (60%) relied on outsourced laboratories for CMV diagnostics, with 82.4% experiencing turnaround times exceeding three days for results. Only 8.6% TP had access to molecular testing for CMV-resistant strains.

**Conclusion:**

This survey reveals substantial variability in CMV diagnosis and management among Brazilian kidney transplant centers, with limited diagnostic access and delays due to reliance on outsourced laboratories. Expanding diagnostic capacity and standardizing guidelines are essential to improving patient outcomes.

## Introduction

Cytomegalovirus (CMV) is the most prevalent opportunistic infection in solid organ transplant (SOT) recipients, contributing significantly to morbidity and mortality^1^. Strategies for CMV prevention, including universal prophylaxis and preemptive antiviral therapy (PET), have demonstrated efficacy in reducing CMV disease^2^. However, implementing these strategies can be challenging, particularly in resource-limited settings where access to key diagnostic tools, such as CMV real-time polymerase chain reaction (qPCR), is restricted^3^. Despite preventive measures, CMV infection remains a concern, affecting up to 50% of high-risk patients (defined as serological mismatch D+/R−) and 17% of moderate-risk patients (defined as CMV-seropositive recipients R+)^4^.

Systematic CMV monitoring is essential for managing SOT recipients, enabling risk prediction, guiding antiviral therapy, and assessing treatment efficacy and resistance^5^. Quantitative PCR for CMV-DNAemia in blood is the preferred diagnostic method due to its high sensitivity and efficiency, improving reliability with an international standard. However, interassay variability remains a challenge, and monitoring practices vary significantly across centers^6,7^. This study aimed to evaluate the practices available for diagnosis and monitoring CMV infection in kidney transplant centers in Brazil, focusing on diagnostic methods, accessibility, and logistical challenges. Additionally, it investigated PET initiation thresholds across risk groups.

## Methods

This study used an electronic questionnaire (Google Forms) to collect data from kidney TP across Brazil. The survey comprised three sections: (1) demographics and centers’ characteristics, (2) CMV diagnostic methods (including sample type, test location, and PET initiation criteria), and (3) access to resistant strain testing and availability of qPCR for non-blood samples.

The questionnaire was emailed to key opinion leaders performing kidney transplantation across 170 centers between August and October 2024, using the Brazilian Society of Organ Transplantation (ABTO) mailing list. Transplant physicians were directly contacted by study authors, and two additional follow-up reminders were sent to enhance response rate. The study was approved by the local Research Ethics Committee, and electronic informed consent was obtained.

To ensure data reliability, only one response per center was analyzed. In cases of multiple responses from the same center, a hierarchical selection process was implemented: (i) preference was given for the program coordinator; (ii) if unavailable, the response from the most experienced professional was selected; and (iii) in the absence of both, the most comprehensive response was chosen. Centers with incomplete responses were contacted to obtain the missing data.

Data were exported to Excel (Microsoft Office, Redmond, WA, USA) and analyzed using SPSS software version 22.0 (IBM Corp., Armonk, NY, USA). Descriptive statistics were performed to summarize results in frequencies and percentages. Partially completed surveys were included, with valid responses used for calculations. Comparative analyses were performed using the Mann-Whitney U test for non-parametric continuous variables, and categorical associations were assessed using the chi-square or Fisher-Freeman-Halton Exact test, as appropriate. A p-value of ≤0.05 was considered statistically significant.

## Results

### Characteristics of Participanting Transplant Programs

Of the 170 kidney TP registered with ABTO, 35 participated in the study, yielding a response rate of 20.6%. The majority of respondents were nephrologists (n = 29; 82.9%), followed by infectious disease specialists (n = 5; 14.3%) and one surgeon (n = 1; 2.9%). Collectively, these centers performed 3,742 kidney transplants in 2023, accounting for 61.9% of the total 6,047 kidney transplants procedures conducted in Brazil that year, according to data from the Brazilian Transplant Registry. Most centers had extensive experience in kidney transplant management, with 82.9% (n = 29) having over 10 years of experience, 11.4% (n = 4) had 5–10 years, and 5.7% (n = 2) had 1–5 years of experience.

**Figure 1:**
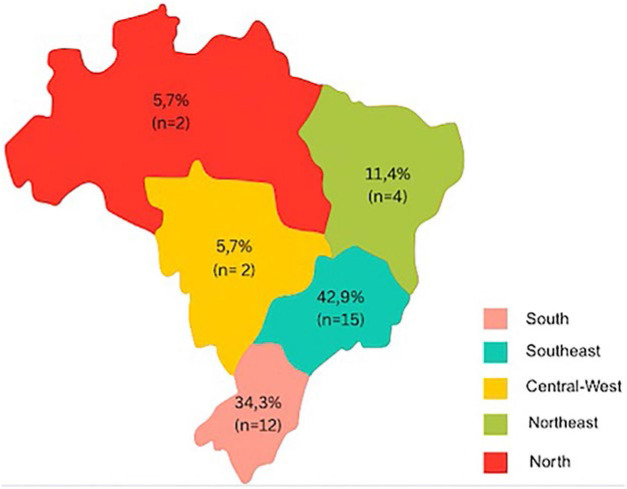
Regional distribution of participating kidney transplant centers in Brazil.

The highest proportion of responses were from the state of Rio Grande do Sul (n = 6; 17.1%), followed by São Paulo (n = 5; 14.3%) and Rio de Janeiro (n = 4; 11.4%). The South and Southeast regions collectively accounted for 77.1% (n = 27) of respondents, reflecting the concentration of transplant activity in these areas (Figure 1).

Regarding healthcare funding, 42.9% of institutions operated exclusively within the public health system (n = 15), 5.7% served only the private sector (n = 2), and 51.4% provided both public and private care (n = 18). Mixed-funding institutions were more prevalent in the South and Southeast regions (65.7%), whereas other regions predominantly offered exclusively public healthcare services (p < 0.001).

The majority of centers reported having institutional protocols for managing CMV infection in kidney transplant patients (n = 33, 94.3%)

### CMV Diagnostic Methods

Most of TP (n = 34; 97.1%) relied on qPCR techniques for monitoring CMV infection, while CMV antigenemia was reported by a single center (2.9%). Commercial qPCR assays were preferred (n = 24; 68.6%) over in-house tests (n = 6; 17.1%), whereas five centers (14.3%) did not specify the assay type. Whole blood (n = 21; 60.0%) and plasma (n = 12; 34.3%) were the main sample types used, with one center (2.9%) using both. CMV viral load was expressed in genomic copies/mL in 17 centers (56.7%) and international units per milliliter (IU/mL) in 13 (43.3%). Twelve centers (n = 12/34; 35.2%) reported using log10 values for PET initiation, with four not specifying whether they referred to copies/mL or IU/mL. Eight centers (23.5%) used a combination of log10 with the integrated value.

Regarding test execution, 60.0% (n = 21) of centers outsourced diagnostic testing, while 40.0% (n = 14) conducted testing on-site. Among those outsourcing CMV qPCR testing, 80.9% (n = 17) reported that outsourcing influenced the turnaround time for obtaining results, with the majority experiencing turnaround times exceeding three days (82.4%, n = 14). Only a small proportion received results within 24 hours (5.9%, n = 1) or within 48 hours (11.8%, n = 2).

Geographic region was not significantly associated with the type of test (commercial vs. in-house, p = 0.549) or test location (on-site vs. outsourced, p = 0.312). Similarly, the size of the centers (based on the number of transplants performed in 2023) was not associated with the type of test or test location (p = 0.657 and p = 0.266, respectively). Table 1 details the diagnostic methods used by participating centers.

**Table 1:** Tests available to diagnose and monitor CMV infection in kidney transplant centers in brazil

Topics surveyed	n (%)
Test used for CMV surveillance (respondents = 35)	
qPCR in plasma	12 (34.3)
qPCR in whole blood	21 (60.0)
CMV antigenemia	1 (2.9)
qPCR in plasma + whole blood	1 (2.9)
QNAT Methods Used (respondents = 35)	
qPCR commercial test	24 (68.6)
In-house diagnostic test	6 (17.1)
Unknown	5 (14.3)
Test Execution Location (respondents = 35)	
Outsourced testing	21 (60.0)
On site testing	14 (40.0)
Impact of Outsourced Testing on Diagnosis Time (respondents = 21)	
Yes	17 (81.0)
No	4 (19.0)
Turnaround time to Result Release (respondents = 17)	
Within 24 hours	1 (5.9)
Within 48 hours	2 (11.8)
3–5 days	5 (29.4)
5–7 days	7 (41.2)
>7 days	2 (11.8)

Abbreviations – CMV, cytomegalovirus; qPCR, real time polymerase chain reaction testing; QNAT, Quantitative Nucleic Acid Amplification Testing.

### CMV Monitoring

**Figure 2:**
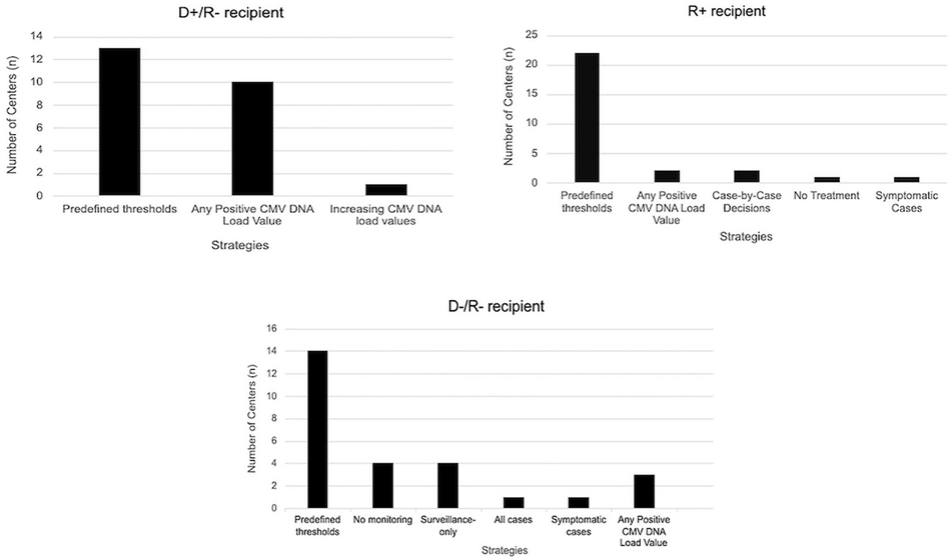
CMV Preemptive Therapy Strategies According to Risk Groups in Kidney Transplant Centers in Brazil (2024).

The criteria for PET initiation varied widely among centers, regardless of whether whole blood or plasma was used. For high-risk patients (D+/R−), 32.2% (n = 10/31) initiated treatment upon detecting any viral load, while 41.9% (n = 13/31) used predefined thresholds. In the R+ group, the majority (n = 22/31, 71.0%;) applied specific cut-off values, whereas in the low-risk group (D−/R−), a smaller proportion (n = 14/31; 45.2%) established thresholds. Variations in PET strategies across different risk groups are detailed in Figure 2, with the corresponding cut-off points outlined below.

Abbreviations – CMV – cytomegalovirus; D+/R− seropositive donor to a seronegative recipient; R+ seropositive recipient; D−/R− seronegative donor to a seronegative recipient.

**High-risk patients (D+/R−):** thresholds ranged from 200 to 2,500 IU/mL in plasma (median: 2,500 IU/mL) and 500 to 2,500 IU/mL in whole blood (median: 1,000 IU/mL). In copies/mL, thresholds varied between 200 and 5,000 in plasma (median: 2,600 copies/mL) and 200 to 7,000 in whole blood (median: 1,250 copies/mL). One center reported a threshold of 5,000 copies/mL for either whole blood or plasma samples.

**Moderate-risk patients (D+ or D-/R+):** thresholds ranged from 2,500 to 5,000 IU/mL in plasma (median: 5,000 IU/mL) and 1,000 to 10,000 IU/mL in whole blood (median: 3,000 IU/mL) while thresholds in terms of copies/mL ranged from 200 to 5,000 in plasma (median: 1,500 copies/mL) and 400 to 7,000 in whole blood (median: 2,500 copies/mL). Two centers reported a threshold of 5,000 copies/mL using both whole blood and plasma samples.

**Low-risk patients (D−/R−):** thresholds varied largely, including 5,000 copies/mL for either plasma or whole blood. Plasma thresholds ranged from 200 to 5,000 IU/mL (median: 5,000 IU/mL), while in whole blood, thresholds varied between 1,000 to 10,000 IU/mL (median: 5,000 IU/mL). In copies/mL, plasma thresholds ranged from 200 to 5,000 (median: 2,600 copies/mL), whereas whole blood thresholds ranged from 200 to 7,000 (median: 1,500 copies/mL).

### Access to Molecular Diagnostics for Detection of CMV-Resistance Strains

Most centers reported lacking access to molecular diagnostics for detecting CMV-resistant strains (n = 28, 80.0%), while 8.6% (n = 3) had access, with two of these centers using genomic sequencing. Another 11.4% (n = 4) were uncertain about the availability of such testing.

### Use of qpcr for Clinical Specimens other than Blood

Access to qPCR testing for CMV varied across centers. Overall, testing was available in 54.3% (n = 19) for cerebrospinal fluid (CSF) samples, 31.4% (n = 11) for bronchoalveolar lavage (BAL) samples, 20.0% (n = 7) for urine samples, 17.1% (n = 6) for fresh biopsies, and 20.0% (n = 7) for bone marrow aspirates. The proportion of centers with access to CMV testing in CSF was identical between those with on-site laboratories and those using external facilities (54.55% vs. 54.55%; p = 1.000). For non-CSF samples, qPCR testing was available in 50.0% of centers with on-site laboratories and in 23.81% of centers relying on external laboratories (p = 0.217).

## Discussion

This survey provides insights into the capacity of kidney transplant centers in Brazil to diagnose and monitor CMV infection. Although the response rate was 20.6%, participating centers accounted for the majority of kidney transplants performed in the country in 2023 (62%), highlighting the national relevance of the findings. This concentration reflects the centralized structure of transplant activity in Brazil. Moreover, the response rate is consistent with that of similar nationwide surveys^3,8,9^.

Our analysis confirms qPCR as the primary CMV monitoring method, with whole blood as the preferred specimen^6^. International guidelines consider whole blood and plasma equally acceptable, emphasizing the importance of consistent specimen selection; whole blood, however, often allows earlier CMV detection and presents higher DNAemia levels than plasma^1,2^. Commercial qPCR was widely used, which may be related to factors such as standardization and sensitivity^10^.

A key limitation of qPCR is the absence of universally defined CMV DNAemia thresholds, as no single consensus value exists for initiating PET across all patient populations^1,11^. In our study, PET initiation criteria varied according to patient risk: among high-risk individuals, therapy was initiated either at any detectable viral load or based on predefined thresholds. In CMV-seropositive recipients (R+), most centers applied a defined viral load threshold for intervention. Although not specifically asked, approximately 20% of centers spontaneously reported using prophylaxis in high-risk patients (D+/R−). In low-risk patients, despite consensus recommendations advising against routine CMV prevention^1^, 45.2% implemented PET cut-off values, while others used no CMV monitoring or prophylaxis. This heterogeneity, further compounded by differences in reporting units (IU/mL vs. copies/mL), specimen types (whole blood vs. plasma), and assay platforms, limits both the interpretation of viral load kinetics and the uniform application of PET thresholds in clinical practice.

Consistent with our findings, a large multicenter European study also demonstrated wide variability in PET criteria, with most centers using a 1,000 IU/mL cut-off in whole blood or plasma, while others initiated therapy at any detectable DNAemia or after unspecified increases in serial measurements.^9^ Such variability may influence clinical outcomes by affecting the timing and appropriateness of therapy initiation, potentially compromising CMV disease control and patient prognosis.

Current international guidelines recommend reporting CMV-DNAemia in log10 IU/mL (with integer values optional), aligned with WHO standards, to enable more consistent interpretation^1^; in our survey, 35.2% of centers reported presenting results in this format. Broader adoption of this practice could facilitate comparability across centers and support the development of more uniform PET thresholds. Taken together, these findings highlight the urgent need for national initiatives to standardize PET criteria—such as technical consensus statements or evidence-based guidelines—in order to reduce inter-center variability and optimize CMV management across transplant programs.

The variability in CMV management observed aligns with the findings reported by Santoro-Lopes *et al*. (2025), which included other types of organ transplants. In their study, 85% of SOT programs in Brazil implemented PET, while 64% used CMV prophylaxis, and 12% relied exclusively on prophylaxis (8). Although our study did not assess the specific antiviral treatments, the lower adoption of CMV prophylaxis in Santoro-Lopes *et al*. likely reflects similar structural constraints, where PET is often preferred due to high cost of valganciclovir, a major barrier to universal prophylaxis.

We identified that the majority of CMV diagnostic programs rely on outsourced laboratories, with long turnaround times (>72h) frequently reported. This dependence often leads to delayed diagnoses, impacting timely decision-making, as early detection is critical for preventing CMV disease progression^12^. In the context of prolonged turnaround times, higher cut-offs may further delay therapy initiation, increasing the risk of progression from asymptomatic DNAemia to clinically significant CMV disease, as well as indirect effects such as acute rejection and opportunistic infection by infectious agents other than CMV. This issue could be mitigated through a more structured system for sample collection and processing, allowing for higher testing volumes and improved efficiency. Additionally, the establishment of regional reference laboratories with the capacity to handle larger test volumes could optimize resource utilization, reduce turnaround times, and enhance patient care.

Rapid CMV detection in BAL fluid, CSF, bone marrow, and fresh tissue is crucial for timely diagnosis and management^13^. Access to CMV qPCR in non-blood samples varied, with CSF being the most available and similarly accessible in both on-site and outsourced laboratories. On-site labs had greater access to other specimens, suggesting that outsourcing may contribute to delays or testing limitations.

CMV resistance remains a significant challenge in KT recipients, particularly among high-risk (D+/R−) patients^4^. Despite its clinical relevance, access to molecular diagnostics for CMV resistance is highly restricted in resource-limited settings. Only 8.6% of centers reported availability, a considerably lower proportion compared to high-income settings, where 43%–79% of centers had access to molecular diagnostic testing^9,14,15^.

This study has limitations, including a low response rate, though the major kidney transplant centers in Brazil participated. Although these centers represent a large share of transplant activity nationwide, the limited number of participants may restrict the external validity of our findings, as practices from smaller or less-resourced centers might not be represented. The regional distribution of responses was also uneven, with higher participation in the Southeast and South and lower participation in the North, potentially introducing non-response bias and further limiting generalizability. Consequently, the actual variability in CMV management across Brazilian transplant programs could be even greater, underscoring the need for broader and more inclusive national surveys.

Furthermore, some gaps remained despite efforts to address missing data. While 94.3% of centers followed institutional protocols, respondents may not fully reflect the actual practices of their institutions. Additionally, self-selection bias from voluntary participation and potential response bias due to question formulation cannot be excluded. The absence of recipient outcome data limits the ability to evaluate the clinical impact of the reported practices. While this limitation is understandable given the scope of our study, future research should aim to combine survey results with clinical endpoints to provide a more comprehensive assessment of CMV management strategies and enhance the applicability of the findings.

This survey reveals considerable heterogeneity in CMV diagnostics and management among Brazilian kidney transplant centers. Limited access to molecular testing and reliance on outsourced laboratories with prolonged turnaround times contribute to diagnostic delays, potentially impacting patient care. Expanded diagnostic capabilities and standardized guidelines are needed to improve clinical outcomes. A national reference center or the Ministry of Health could help implement and ensure adherence to standardized laboratory protocols nationwide.

## Data Availability

The datasets generated and/or analyzed during the current study are not publicly available due to ethical, legal, and privacy restrictions, but are available from the corresponding author on reasonable request.
