# Supplementary material for: Evaluation of practices for the diagnosis and monitoring of cytomegalovirus infection in kidney transplant recipients in Brazil
Source: J Bras Nefrol. 2025 Nov 28;48(2):e20250201. doi: 10.1590/2175-8239-JBN-2025-0201en (PMC12674907; doi:10.1590/2175-8239-JBN-2025-0201en)
Supplement: Translation

# Translation: Avaliação das práticas para o diagnóstico e monitoramento da infecção por citomegalovírus em receptores de transplante renal no Brasil

**DOI:** 10.1590/2175-8239-JBN-2025-0201pt

**Authors:** Nayla Azanki Hatem, Elizete Keitel, Guilherme Santoro-Lopes, Ligia Camera Pierrotti, Alessandro Comarú Pasqualotto

**Affiliations:** 1 Universidade Federal de Ciências da Saúde de Porto Alegre, Programa de Pós-Graduação em Patologia, Porto Alegre, RS, Brasil.; 2 Santa Casa de Misericórdia de Porto Alegre, Porto Alegre, RS, Brasil.; 3 Universidade Federal do Rio de Janeiro, Rio de Janeiro, RJ, Brasil.; 4 Universidade de São Paulo, São Paulo, SP, Brasil.

**Keywords:** Citomegalovírus, Diagnóstico, Terapia, Pesquisa, Transplante renal

## Abstract

**Introdução:**

O citomegalovírus (CMV) permanece um desafio significativo no transplante renal. Apesar das estratégias antivirais profiláticas e preemptivas, as práticas clínicas variam amplamente. Este estudo avaliou práticas de diagnóstico e monitoramento do CMV em centros brasileiros de transplante renal, concentrando-se no acesso a ferramentas diagnósticas, limites terapêuticos e barreiras logísticas.

**Métodos:**

Realizou-se pesquisa eletrônica nacional entre agosto-outubro de 2024, direcionada a todos os programas de transplante (PT) renal registrados na Associação Brasileira de Transplante de Órgãos (ABTO).

**Resultados:**

Participaram 35 PT (taxa de resposta: 20,6%), representando 62% dos transplantes renais realizados no Brasil em 2023. Embora a maioria dos centros possuísse protocolos de manejo do CMV (94,3%), observou-se variabilidade significativa no início da terapia preemptiva (TPE). Entre pacientes de alto risco (D+/R−), 41,9% seguiram limites pré-definidos. Aplicaram-se valores de corte específicos em 71,0% dos pacientes R+ e em 45,2% do grupo baixo risco (D−/R−). PCR quantitativa foi o principal método diagnóstico (97,1%), com sangue total (60%) e plasma (34,3%) como tipos de amostra preferidos. Uma proporção significativa dos PT (60%) dependia de laboratórios terceirizados para diagnóstico de CMV, com 82,4% apresentando tempo de retorno dos resultados superior a três dias. Apenas 8,6% dos PT tinham acesso a testes moleculares para cepas de CMV resistentes.

**Conclusão:**

Esta pesquisa revela variabilidade substancial no diagnóstico e manejo do CMV entre centros brasileiros de transplante renal, com acesso limitado ao diagnóstico e atrasos decorrentes da dependência de laboratórios terceirizados. Ampliar a capacidade diagnóstica e padronizar diretrizes são essenciais para melhoria dos desfechos dos pacientes.

## Introdução

O citomegalovírus (CMV) é a infecção oportunista mais prevalente em receptores de transplante de órgãos sólidos (TOS), contribuindo de forma significativa para a morbidade e mortalidade^1^. Estratégias para a prevenção do CMV, incluindo profilaxia universal e terapia antiviral preemptiva (TPE), demonstraram eficácia na redução da doença por CMV^2^. No entanto, a implementação dessas estratégias pode ser desafiadora, especialmente em contextos com recursos limitados, onde o acesso a ferramentas diagnósticas essenciais, como a reação em cadeia da polimerase em tempo real (qPCR) para CMV, é restrito^3^. Apesar das medidas preventivas, a infecção por CMV continua sendo motivo de preocupação, afetando até 50% dos pacientes de alto risco (definidos como incompatibilidade sorológica D+/R−) e 17% dos pacientes de risco moderado (definidos como receptores soropositivos para CMV [R+])^4^.

O monitoramento sistemático do CMV é essencial para o manejo de receptores de TOS, permitindo a predição de riscos, orientando a terapia antiviral e avaliando a eficácia e a resistência do tratamento^5^. A PCR quantitativa para detecção de DNA do CMV (CMV-DNAemia) no sangue é o método diagnóstico de escolha devido à sua alta sensibilidade e eficiência, aumentando a confiabilidade com um padrão internacional. No entanto, a variabilidade entre ensaios ainda representa um desafio, e as práticas de monitoramento variam significativamente entre os centros^6,7^. Este estudo teve como objetivo avaliar as práticas disponíveis para o diagnóstico e monitoramento da infecção por CMV em centros de transplante renal no Brasil, com foco nos métodos diagnósticos, acessibilidade e desafios logísticos. Além disso, investigou os limites para o início da TPE entre os grupos de risco.

## Métodos

O presente estudo utilizou um questionário eletrônico (*Google Forms*) para coletar dados sobre PT renal em todo o Brasil. A pesquisa compreendeu três seções: (1) dados demográficos e características dos centros; (2) métodos diagnósticos para CMV (incluindo tipo de amostra, local do teste e critérios para início da TPE); e (3) acesso a testes para detecção de cepas resistentes e disponibilidade de qPCR para amostras não sanguíneas.

O questionário foi enviado por e-mail a importantes líderes de opinião responsáveis por transplantes renais em 170 centros, entre agosto e outubro de 2024, utilizando a lista de endereços eletrônicos vinculada à Associação Brasileira de Transplante de Órgãos (ABTO). Os médicos transplantadores foram contatados diretamente pelos autores do estudo e dois lembretes adicionais foram enviados para aumentar a taxa de resposta. O estudo foi aprovado pelo Comitê de Ética em Pesquisa local, e foi obtido consentimento informado eletrônico.

Para garantir a confiabilidade dos dados, foi analisada somente uma resposta por centro. Nos casos em que houve múltiplas respostas provenientes do mesmo centro, implementou-se um processo hierárquico de seleção: (i) foi dada preferência ao coordenador do programa; (ii) na ausência deste, foi selecionada a resposta do profissional com maior experiência; e (iii) na ausência de ambos, foi escolhida a resposta mais abrangente. Os centros com respostas incompletas foram contatados com o objetivo de obter os dados faltantes.

Os dados foram exportados para o Excel (*Microsoft Office*, Redmond, WA, EUA) e analisados no software SPSS versão 22.0 (*IBM Corp.*, Armonk, NY, EUA). Estatísticas descritivas sintetizaram os resultados em frequências e porcentagens. Pesquisas parcialmente preenchidas foram incluídas, com respostas válidas utilizadas para cálculos. Foram realizadas análises comparativas por meio do teste U de *Mann-Whitney* para variáveis contínuas não paramétricas, e avaliaram-se as associações categóricas por meio do teste qui-quadrado ou do teste exato de *Fisher-Freeman-Halton*, conforme apropriado. Um valor de p ≤ 0,05 foi considerado estatisticamente significativo.

## Resultados

### Características dos Programas de Transplante Participantes

Dos 170 PT renal registrados na ABTO, 35 participaram do estudo, o que gerou uma taxa de resposta de 20,6%. A maioria dos respondentes era composta por nefrologistas (n = 29; 82,9%), seguidos por especialistas em doenças infecciosas (n = 5; 14,3%) e um cirurgião (n = 1; 2,9%). Em conjunto, esses centros realizaram 3.742 transplantes renais em 2023, representando 61,9% do total de 6.047 procedimentos realizados no Brasil naquele ano, de acordo com dados do Registro Brasileiro de Transplantes. A maioria dos centros apresentava ampla experiência no manejo de transplantes renais, com 82,9% (n = 29) tendo mais de 10 anos de experiência, 11,4% (n = 4) entre 5 e 10 anos e 5,7% (n = 2) entre 1 e 5 anos de experiência.

A maior proporção de respostas veio do estado do Rio Grande do Sul (n = 6; 17,1%), seguido por São Paulo (n = 5; 14,3%) e Rio de Janeiro (n = 4; 11,4%). As regiões Sul e Sudeste, juntas, representaram 77,1% (n = 27) dos respondentes, refletindo a concentração da atividade transplantadora nessas áreas. (Figura 1).

**Figura 1:** Distribuição regional dos centros de transplante renal participantes no Brasil.

Em relação ao financiamento dos serviços de saúde, 42,9% das instituições operavam exclusivamente dentro do sistema público de saúde (n = 15), 5,7% atendiam apenas ao setor privado (n = 2) e 51,4% prestavam serviços tanto públicos quanto privados (n = 18). As instituições de financiamento misto foram mais prevalentes nas regiões Sul e Sudeste (65,7%), enquanto as demais regiões apresentaram predominância de serviços de saúde exclusivamente públicos (p < 0,001).

A maioria dos centros relatou possuir protocolos institucionais para o manejo da infecção por CMV em pacientes submetidos a transplante renal (n = 33; 94,3%).

### Métodos Diagnósticos para CMV

A maioria dos PT (n = 34; 97,1%) baseou-se em técnicas de qPCR para o monitoramento da infecção por CMV, enquanto a antigenemia para CMV foi relatada por um único centro (2,9%). Os ensaios comerciais de qPCR foram preferidos (n = 24; 68,6%) em relação aos testes *in-house* (n = 6; 17,1%), enquanto cinco centros (14,3%) não especificaram o tipo de ensaio empregado. Os principais tipos de amostra utilizados foram sangue total (n = 21; 60,0%) e plasma (n = 12; 34,3%), com um centro (2,9%) relatando o uso de ambos. A carga viral de CMV foi expressa em cópias genômicas/mL em 17 centros (56,7%), e em unidades internacionais por mililitro (UI/mL) em 13 (43,3%). Doze centros (n = 12/34; 35,2%) relataram o uso de valores log_10_ para o início da TPE, com quatro deles não especificando se os valores se referiam a cópias/mL ou UI/mL. Oito centros (23,5%) empregaram uma combinação de log_10_ com o valor integrado.

Com relação à execução dos testes, 60,0% (n = 21) dos centros terceirizavam os testes diagnósticos, enquanto 40,0% (n = 14) realizavam os testes internamente. Entre aqueles que terceirizavam os testes de qPCR para CMV, 80,9% (n = 17) relataram que a terceirização influenciava o tempo de resposta, com a maioria apresentando prazos superiores a três dias (82,4%; n = 14) para obtenção dos resultados. Apenas uma pequena proporção recebeu os resultados dentro de 24 horas (5,9%; n = 1) ou dentro de 48 horas (11,8%; n = 2).

A região geográfica não apresentou associação significativa com o tipo de teste (comercial vs. desenvolvido internamente; p = 0,549) ou com o local de realização (no local vs. terceirizado; p = 0,312). De forma semelhante, o tamanho dos centros (com base no número de transplantes realizados em 2023) não foi associado ao tipo de teste ou local de execução (p = 0,657 e p = 0,266, respectivamente). A Tabela 1 detalha os métodos diagnósticos utilizados pelos centros participantes.

**Tabela 1:** Testes disponíveis para o diagnóstico e monitoramento da infecção por CMV em centros de transplante renal no brasil

Tópicos abordados	n (%)
Teste utilizado para vigilância do CMV (respondentes = 35)	
qPCR no plasma	12 (34,3)
qPCR no sangue total	21 (60,0)
Antigenemia para CMV	1 (2,9)
qPCR no plasma + sangue total	1 (2,9)
Métodos QNAT utilizados (respondentes = 35)
Teste qPCR comercial	24 (68,6)
Teste diagnóstico interno	6 (17,1)
Desconhecido	5 (14,3)
Local de Execução do Teste (respondentes = 35)
Terceirização dos testes	21 (60,0)
Testes realizados internamente	14 (40,0)
Impacto da Terceirização de Testes no Tempo de Diagnóstico (respondentes = 21)	
Sim	17 (81,0)
Não	4 (19,0)
Tempo de Resposta até a Divulgação dos Resultados (respondentes = 17)	
Dentro de 24 horas	1 (5,9)
Dentro de 48 horas	2 (11,8)
3–5 dias	5 (29,4)
5–7 dias	7 (41,2)
>7 dias	2 (11,8)

Abreviaturas – CMV, citomegalovírus; qPCR, teste de reação em cadeia da polimerase em tempo real; QNAT, teste quantitativo de amplificação de ácido nucleico.

### Monitoramento do CMV

Os critérios para o início da TPE variaram amplamente entre os centros, independentemente do tipo de amostra utilizada (sangue total ou plasma). Para pacientes de alto risco (D+/R−), 32,2% (n = 10/31) iniciaram o tratamento após a detecção de qualquer carga viral, enquanto 41,9% (n = 13/31) utilizaram limites pré-definidos. No grupo R+, a maioria (n = 22/31; 71,0%) aplicou valores de corte específicos, enquanto no grupo de baixo risco (D−/R−), uma proporção menor (n = 14/31; 45,2%) estabeleceu limites. As variações nas estratégias de TPE entre os diferentes grupos de risco estão detalhadas na Figura 2, com os pontos de corte correspondentes descritos abaixo.

**Figura 2:** Estratégias de Terapia Preemptiva para CMV de acordo com os Grupos de Risco em Centros de Transplante Renal no Brasil (2024).

Abreviaturas – CMV – citomegalovírus; D+/R− doador soropositivo para receptor soronegativo; R+ receptor soropositivo; D−/R− doador soronegativo para receptor soronegativo.

**Pacientes de alto risco (D+/R−):** os limites variaram de 200 a 2.500 UI/mL no plasma (mediana: 2.500 UI/mL) e de 500 a 2.500 UI/mL no sangue total (mediana: 1.000 UI/mL). Quando expressos em cópias/mL, os limites variaram entre 200 e 5.000 no plasma (mediana: 2.600 cópias/mL) e 200 a 7.000 no sangue total (mediana: 1.250 cópias/mL). Um centro relatou um limite de 5.000 cópias/mL tanto para amostras de sangue total quanto de plasma.

**Pacientes de risco moderado (D+ ou D-/R+):** os limites variaram de 2.500 a 5.000 UI/mL no plasma (mediana: 5.000 UI/mL) e de 1.000 a 10.000 UI/mL no sangue total (mediana: 3.000 UI/mL), enquanto os limites em termos de cópias/mL variaram entre 200 e 5.000 no plasma (mediana: 1.500 cópias/mL) e de 400 a 7.000 no sangue total (mediana: 2.500 cópias/mL). Dois centros relataram um limite de 5.000 cópias/mL usando ambas as amostras de sangue total e plasma.

**Pacientes de baixo risco (D−/R−):** os limites apresentaram ampla variação, incluindo 5.000 cópias/mL para plasma ou sangue total. Os limites plasmáticos variaram de 200 a 5.000 IU/mL (mediana: 5.000 IU/mL), enquanto no sangue total, os limites variaram entre 1.000 e 10.000 IU/mL (mediana: 5.000 IU/mL). Em cópias/mL, os limites plasmáticos variaram entre 200 e 5.000 (mediana: 2.600 cópias/mL), enquanto os limites no sangue total variaram entre 200 e 7.000 (mediana: 1.500 cópias/mL).

### Acesso A Diagnósticos Moleculares para Detecção de Cepas de CMV Resistentes

A maioria dos centros relatou não ter acesso a diagnósticos moleculares para a detecção de cepas de CMV resistentes (n = 28; 80,0%), enquanto 8,6% (n = 3) relataram ter acesso, com dois desses centros utilizando sequenciamento genômico. Outros 11,4% (n = 4) estavam incertos quanto à disponibilidade desses testes.

### Uso de qpcr para Amostras Clínicas Diferentes das de Sangue

O acesso ao teste de qPCR para CMV variou entre os centros. De modo geral, o exame estava disponível em 54,3% (n = 19) para amostras de líquido cefalorraquidiano (LCR), 31,4% (n = 11) para amostras de lavado broncoalveolar (LBA), 20,0% (n = 7) para amostras de urina, 17,1% (n = 6) para biópsias a fresco e 20,0% (n = 7) para aspirados de medula óssea. A proporção de centros com acesso a testes para CMV no LCR foi idêntica entre aqueles com laboratórios próprios e aqueles que utilizavam instalações externas (54,55% vs. 54,55%; p = 1,000). Para amostras não provenientes do LCR, o teste de qPCR estava disponível em 50,0% dos centros com laboratórios internos e em 23,81% dos centros que dependiam de laboratórios terceirizados (p = 0,217).

## Discussão

Esta pesquisa fornece *insights* sobre a capacidade dos centros de transplante renal no Brasil de diagnosticar e monitorar a infecção por CMV. Embora a taxa de resposta tenha sido de 20,6%, os centros participantes foram responsáveis pela maioria dos transplantes renais realizados no país em 2023 (62%), destacando a relevância nacional dos achados. Essa concentração reflete a estrutura centralizada da atividade transplantadora no país. Além disso, a taxa de resposta é consistente com a de pesquisas semelhantes realizadas em âmbito nacional^3,8,9^.

Nossa análise confirma a qPCR como o principal método de monitoramento do CMV, sendo o sangue total a amostra de preferência^6^. As diretrizes internacionais consideram o sangue total e o plasma igualmente aceitáveis, enfatizando a importância da seleção consistente de amostras; entretanto, o sangue total geralmente permite a detecção mais precoce do CMV e apresenta níveis mais elevados de DNAemia em comparação ao plasma^1,2^. A qPCR comercial foi amplamente utilizada, o que pode estar relacionado a fatores como padronização e sensibilidade^10^.

Uma limitação importante da qPCR é a ausência de limites universalmente definidos para a DNAemia do CMV, uma vez que não existe um valor de consenso único para o início da TPE em todas as populações de pacientes^1,11^. Em nosso estudo, os critérios para o início da TPE variaram de acordo com o nível de risco do paciente: entre os indivíduos de alto risco, a terapia foi iniciada a partir de qualquer carga viral detectável ou com base em limites predefinidos. Em receptores soropositivos para CMV (R+), a maioria dos centros aplicou um limite definido de carga viral para intervenção. Embora não tenha sido especificamente solicitado, aproximadamente 20% dos centros relataram, de forma espontânea, o uso de profilaxia em pacientes de alto risco (D+/R−). Em pacientes de baixo risco, apesar de recomendações consensuais desaconselharem a prevenção rotineira do CMV^1^, 45,2% implementaram valores de corte para TPE, enquanto outros não utilizaram monitoramento ou profilaxia para CMV. Essa heterogeneidade, agravada ainda mais pelas diferenças nas unidades de notificação (UI/mL vs. cópias/mL), nos tipos de amostra (sangue total vs. plasma) e nas plataformas de ensaio, limita tanto a interpretação da cinética da carga viral quanto a aplicação uniforme dos limites para TPE na prática clínica.

Em consonância com nossos achados, um grande estudo multicêntrico europeu também demonstrou ampla variabilidade nos critérios da TPE, com a maioria dos centros utilizando um ponto de corte de 1.000 UI/mL no sangue total ou plasma, enquanto outros iniciaram a terapia a partir de qualquer DNAemia detectável ou após aumentos não especificados em medições seriadas^9^. Essa variabilidade pode influenciar os desfechos clínicos, afetando o momento e a adequação do início da terapia, o que potencialmente compromete o controle da doença por CMV e o prognóstico do paciente.

As diretrizes internacionais atuais recomendam que a DNAemia do CMV seja relatada em valores log_10_ IU/mL (com valores integrais opcionais), em conformidade com os padrões da OMS, para permitir uma interpretação mais consistente^1^. Em nossa pesquisa, 35,2% dos centros relataram apresentar resultados nesse formato. A adoção mais ampla dessa prática poderia facilitar a comparabilidade entre os centros e apoiar o desenvolvimento de limites de TPE mais uniformes. Em conjunto, esses achados destacam a necessidade urgente de iniciativas nacionais voltadas à padronização dos critérios de TPE—tais como declarações de consenso técnico ou diretrizes baseadas em evidências—com o objetivo de reduzir a variabilidade entre centros e otimizar o manejo do CMV em todos os programas de transplante.

A variabilidade observada no manejo do CMV está em consonância com os achados relatados por Santoro-Lopes *et al*. (2025), que incluíram outros tipos de transplantes de órgãos. No referido estudo, 85% dos programas de TOS no Brasil implementaram a TPE, enquanto 64% utilizaram a profilaxia para CMV e 12% basearam-se exclusivamente na profilaxia (8). Embora nosso estudo não tenha avaliado tratamentos antivirais específicos, a menor adoção da profilaxia para CMV observada em Santoro-Lopes *et al*. provavelmente reflete restrições estruturais semelhantes, nas quais a TPE é frequentemente preferida devido ao alto custo do valganciclovir, o que constitui uma grande barreira à profilaxia universal.

Identificamos que a maioria dos programas de diagnóstico de CMV depende de laboratórios terceirizados, sendo frequentemente relatados longos prazos de entrega (>72 horas). Essa dependência, muitas vezes, leva a atrasos nos diagnósticos, com impacto na tomada de decisão oportuna, uma vez que a detecção precoce é fundamental para prevenir a progressão da doença por CMV^12^. No contexto de tempos de resposta prolongados, valores de corte mais elevados podem atrasar ainda mais o início da terapia, aumentando o risco de progressão de DNAemia assintomática para doença por CMV clinicamente significativa, bem como efeitos indiretos, como rejeição aguda e infecção oportunista por agentes infecciosos que não o CMV. Esta questão poderia ser mitigada por meio de um sistema mais estruturado para coleta e processamento de amostras, que permitisse um volume maior de testes e aumentasse a eficiência. Além disso, o estabelecimento de laboratórios de referência regionais com capacidade para lidar com volumes maiores de testes poderia otimizar a utilização de recursos, reduzir os tempos de resposta e melhorar o cuidado ao paciente.

A detecção rápida do CMV no LBA, LCR, medula óssea e tecidos frescos é crucial para o diagnóstico e o manejo oportunos^13^. O acesso à qPCR para CMV em amostras não sanguíneas variou, sendo o LCR o material mais disponível e igualmente acessível tanto em laboratórios internos quanto terceirizados. Os laboratórios internos tiveram maior acesso a outras amostras, sugerindo que a terceirização pode contribuir para atrasos ou limitações nos testes.

A resistência do CMV continua sendo um desafio significativo em receptores de TR, particularmente entre pacientes de alto risco (D+/R−)^4^. Apesar de sua relevância clínica, o acesso a diagnósticos moleculares de resistência do CMV é altamente restrito em ambientes com recursos limitados. Somente 8,6% dos centros relataram disponibilidade, uma proporção consideravelmente menor em comparação a cenários de alta renda, onde 43% a 79% dos centros tinham acesso a testes moleculares de diagnóstico^9,14,15^.

Este estudo apresenta limitações, incluindo uma baixa taxa de resposta, embora os principais centros de transplante renal do Brasil tenham participado. Apesar de esses centros representarem grande parte da atividade transplantadora em todo o país, o número limitado de participantes pode restringir a validade externa de nossos achados, uma vez que as práticas adotadas por centros menores ou com menos recursos podem não estar representadas. A distribuição regional das respostas também foi desigual, com maior participação nas regiões Sudeste e Sul e menor representatividade da região Norte, o que pode ter introduzido um viés de não resposta e limitado ainda mais a generalização dos resultados. Consequentemente, a variabilidade real no manejo do CMV entre os programas de transplante brasileiros pode ser ainda maior, ressaltando a necessidade de pesquisas nacionais mais amplas e inclusivas.

Além disso, algumas lacunas permaneceram, apesar dos esforços para suprir os dados ausentes. Embora 94,3% dos centros seguissem os protocolos institucionais, os respondentes podem não refletir totalmente as práticas reais de suas instituições. Ademais, não se pode excluir o viés de autosseleção decorrente da participação voluntária e o potencial viés de resposta devido à formulação das questões. A ausência de dados sobre os desfechos dos receptores limita a capacidade de avaliar o impacto clínico das práticas relatadas. Embora essa limitação seja compreensível, dado o escopo do presente estudo, pesquisas futuras devem ter como objetivo combinar os resultados dos levantamentos com os desfechos clínicos, a fim de fornecer uma avaliação mais abrangente das estratégias de manejo do CMV e aprimorar a aplicabilidade dos achados.

Esta pesquisa revela uma heterogeneidade considerável no diagnóstico e no manejo do CMV entre os centros brasileiros de transplante renal. O acesso limitado a testes moleculares e a dependência de laboratórios terceirizados com prazos de entrega prolongados contribuem para atrasos no diagnóstico, podendo afetar o cuidado ao paciente. São necessárias capacidades diagnósticas ampliadas e diretrizes padronizadas para melhorar os desfechos clínicos. Um centro de referência nacional ou o próprio Ministério da Saúde poderiam auxiliar na implementação e garantir a adesão a protocolos laboratoriais padronizados em âmbito nacional.

## Data Availability

Os conjuntos de dados gerados e/ou analisados durante o presente estudo não estão disponíveis publicamente devido a restrições éticas, legais e de privacidade, mas podem ser obtidos junto ao autor correspondente mediante solicitação razoável.
